# Predictive factors of ergonomic behaviors based on social cognitive theory among women workers on assembly lines: application of Bayesian networks

**DOI:** 10.1186/s12891-023-07021-5

**Published:** 2023-11-30

**Authors:** Zakieh Sadat Hosseini, Sedigheh Sadat Tavafian, Omran Ahmadi, Reza Maghbouli

**Affiliations:** 1 Department of Health Education, Faculty of Medical Sciences, Tarbiat Modares University, Tehran, Iran; 2 Department of Occupational Health, Faculty of Medical Sciences, Tarbiat Modares University, Tehran, Iran; 3 School of Medicine, Hasheminejad Hospital, Iran University of Medical Sciences, Tehran, Iran

**Keywords:** Ergonomic behaviors, Bayesian networks, Assembly line workers, Social cognitive theory

## Abstract

**Background:**

This study focuses on identifying the key factors associated with ergonomic behaviors (ERBE) among women workers on assembly lines (WwAL) to prevent musculoskeletal disorders (MSDs) caused by repetitive motions and unfavorable body postures. To achieve this objective, this study employed Bayesian networks (BN) analysis based on social cognitive theory (SCT).

**Methods:**

A cross-sectional study was conducted to examine the predictive factors of ERBE among 250 WwAL from six different industries located in Neyshabur, a city in northeastern Iran. The study used a two-stage cluster sampling method for participant selection and self-report questionnaires to collect data on demographic characteristics, variables associated with SCT, ERBE, and the standard Nordic questionnaire. The collected data were analyzed using Netica and SPSS version 21, which involved statistical analyses such as independent t-tests, Pearson correlation, and ANOVA tests at a significance level of p < 0.05. BN analysis was conducted to identify the important factors that impact ERBE.

**Results:**

The majority of individuals reported experiencing chronic pain in their back, neck, and shoulder areas. Engaging in physical activity, consuming dairy products, and attaining a higher level of education were found to be significantly associated with the adoption of ERBE p < 0.05. Among the various SCT constructs, observational learning, intention, and social support demonstrated the highest levels of sensitivity towards ERBE, with scores of 4.08, 3.82, and 3.57, respectively. However, it is worth noting that all SCT constructs exhibited a certain degree of sensitivity towards ERBE.

**Conclusions:**

The research findings demonstrate that all constructs within SCT are effective in identifying factors associated with ERBE among WwAL. The study also highlights the importance of considering education levels and variables related to healthy lifestyles when promoting ERBE in this specific population.

## Background

Musculoskeletal disorders (MSDs) are not only one of the most common work-related health problems reported in the world but also a prevalent occupational health concern among women in specific occupations [1, 2]. Work-related musculoskeletal disorders (WMSD) commonly present with discomfort, sensitivity, decreased strength, inflammation, and loss of sensation [3]. MSDs can lead to mobility and functionality limitations, ultimately impacting work performance. Additionally, these disorders can have wider social and economic implications, including absenteeism, decreased productivity, and higher healthcare expenses [4].

MSDs can be attributed to a range of factors comprising ergonomic, physical, chemical, and psychological elements. These factors encompass aspects such as incorrect posture, repetitive motions, rapid work pace, non-ergonomic tools, unsuitable workstations, and exposure to certain equipment during work [5, 6]. Assembly lines represent a profession encompassing numerous risk factors associated with MSDs, and extensive research has indicated a notable prevalence of such disorders among individuals employed in assembly line roles [7]. Women account for 60% of all MSD cases, which is a concerning trend for working women who face unique challenges in balancing work and family responsibilities [8].

Utilizing preventive measures for MSDs is essential in managing the considerable direct and indirect expenses associated with WMSD [9]. To mitigate the risk of MSDs among workers, it is advisable to implement ergonomic interventions such as training in proper posture, incorporating stretching exercises, utilizing personal protective equipment, and actively promoting healthy lifestyles [10–13].

**Fig. 1:**
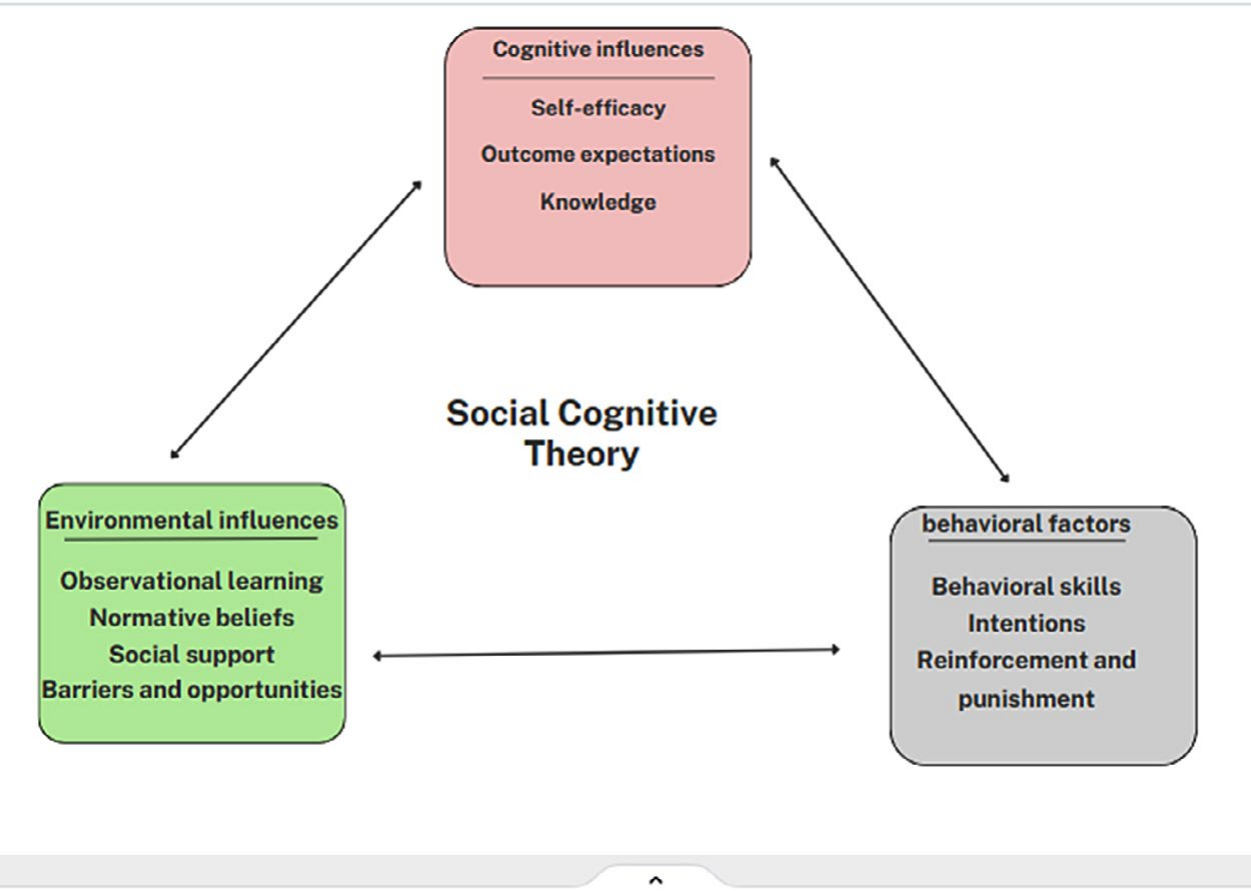
Constructs of social cognitive theory

Understanding individuals’ beliefs and perceptions regarding factors that influence behavior can assist in the creation of impactful interventions aimed at fostering healthy behavior [14]. SCT is a theoretical framework that takes into account various individual, environmental, and cognitive factors that impact behavior. It elucidates how individuals acquire behavioral patterns through interactions with their surroundings, personal characteristics, and cognitive processes [15]. Personal cognitive factors, Socio-environmental factors, and behavioral factors are the three interacting factors involved in the application of SCT to health problems. Personal cognitive factors include self-efficacy, outcome expectations, and knowledge. Socio-environmental factors include observational learning, normative beliefs, social support, opportunities, and barriers. Behavioral factors include behavioral skills, intentions, and reinforcement. SCT suggests that modifying these three factors can achieve the prevention of morbidity and mortality through the development of healthy behaviors and the reduction of unhealthy behaviors [16] (Fig. 1).

Bayesian networks (BN), also referred to as Bayesian belief networks and belief networks, utilize directed acyclic graphs (DAGs) to represent variables. In these graphs, nodes represent the variables, and edges indicate their direct probabilistic dependencies. The field of medicine extensively employs BN due to their interpretability and their facilitation of inference [17]. A BN model demonstrates superior performance compared to neural networks and logistic regression models and effectively elucidates causal relationships between variables [18].

WMSDs have a significant impact on workers’ health and work capacity, resulting in absenteeism, disability pensions, reduced productivity, functional limitations, and disruption of women’s social roles. Therefore, addressing this issue is crucial, as it is recognized as a major challenge in the workplace [19].

In promoting health within the primary healthcare system, health education plays a vital role. Hence, it is crucial for researchers to identify predictors of ergonomic behaviors, particularly for female assembly line workers, to develop more effective interventions [20]. This approach can lead to clinical improvements and advance scientific practice and research.

Moreover, health, safety, and environment (HSE) experts in the industry can contribute significantly to the healthcare system by utilizing the effective constructs of this theory in applying ergonomic behaviors.

Previous studies that investigated the effectiveness of ergonomic behaviors (ERBE) have only used non-industrial environments [21–23], while research on MSDs in industrial workers has not incorporated educational theories [24, 25]. Although some studies have suggested that SCT could improve preventive measures for WMSDs [26, 27], its ability to predict ergonomic behaviors among women workers on assembly lines (WwAL), who are exposed multiple job-related risks, has not been investigated [28, 29]. To the best of our understanding, no research has been conducted using BN to address this topic. The objective of this research was to utilize BN analysis in accordance with the principles of SCT to pinpoint the various factors that impact ERBE.

## Methods

### Study design and setting

From February 11 to March 28, 2023, a cross-sectional study was conducted in Neyshabur, a city located in the northeastern region of Iran. The study focused on women employed in industrial assembly lines.

### Sampling method and sample size

The study employed a two-stage cluster sampling approach to select participants. In the initial stage, a list was compiled of all the electronic industries (a total of 6 industries) with assembly lines where women were employed. Subsequently, a random sampling lottery method was utilized to select eligible women from each industry in proportion to the number of assembly line workers, ensuring an equal representation across industries.

Taking into account an average total of approximately 500 female workers in assembly lines, the sample size was determined to be 217 individuals using Cochran’s formula and considering a type I error of 0.05. However, factoring in a potential dropout rate of 15%, the estimated sample size was adjusted to 250 individuals.

### Eligibility criteria

The inclusion criteria for this study encompassed providing consent to participate, being employed in the assembly line, having an age of over 20 years, and possessing the ability to read and write. On the other hand, no specific exit criteria were taken into account for this research.

### Instruments and measures

The study utilized two questionnaires: the Nordic standard questionnaire, which allows each woman to report pain in all nine body regions simultaneously, enabling respondents to aptly express the complexity of their musculoskeletal experiences; and the Ergonomic Behaviors Evaluation Tool (EBET) questionnaire, specifically designed to assess ERBE of WwAL based on SCT. The reliability and validity of the EBET questionnaire were assessed through comprehensive psychometric analysis, which demonstrated favorable psychometric properties. However, the reference to the original source of the questionnaire is currently under review and can be found in the citation information. The questionnaire exhibited an average CVR of 0.92, a CVI of 0.97, and an overall Cronbach’s alpha coefficient of 0.79. Exploratory factor analysis successfully identified all dimensions of the questionnaire, explaining 65.25% of the variance, and confirmatory factor analysis demonstrated a good fit of the model. The psychometric properties of this tool were confirmed in previous study [30].

### Demographic characteristics

All the women in our study were employed on the assembly lines of the electronics industry, where their work was characterized by repetitive movements, the frequent need to adopt non-optimal postures, and extended periods of sitting during their tasks. Demographic questions, covering age, height, weight, educational status, marital status, work experience, physical activity status, dairy consumption status, and financial status. When choosing the demographic variables for this research, we took into account factors that have previously been identified as potential influencers of musculoskeletal disorders and ergonomic practices which supported by pertinent literature [2, 8, 12, 31, 32]. Participants’ income levels were classified as ‘well,‘ ‘moderate,‘ or ‘poor,‘ based on their subjective evaluation of their financial well-being obtained through the questionnaire. This categorization considers not only the participant’s personal income but also their family’s economic background, providing a holistic perspective on their financial status.

### SCT variables

The researcher-made questionnaire aimed to evaluate ERBE based on SCT constructs and consisted of 11 dimensions, including outcome expectations, normative beliefs, perceived barriers, social support, observational learning, reinforcement, behavioral skills, self-efficacy, intention, and knowledge. Respondents used a 5-point Likert scale to indicate their opinions, and data collection was done through self-reporting methods [30].

### Behavior measurement

The behavior section included questions assessing the engagement in stretching exercises and the adherence to proper body posture during work. Participants rated their responses on a 3-point Likert scale, with options ranging from never to always, assigned values of 1 to 3 points, respectively.

### Data collection

The questionnaires were distributed to WwAL based on their availability during a 20-minute break time, and they were completed through self-reporting. Prior to completing the questionnaire, the researcher provided an explanation of the study’s purpose and the questionnaire’s structure. During the questionnaire completion, the researcher was present in the environment to offer guidance if any difficulties arose. Following the completion of the questionnaires, team members were assigned the task of quality control, reviewing the questionnaires for accuracy and completeness. Ultimately, after removing any incompletely filled-out questionnaires, a total of 250 questionnaires were included in the study.

### Bayesian networks

**Fig. 2:**
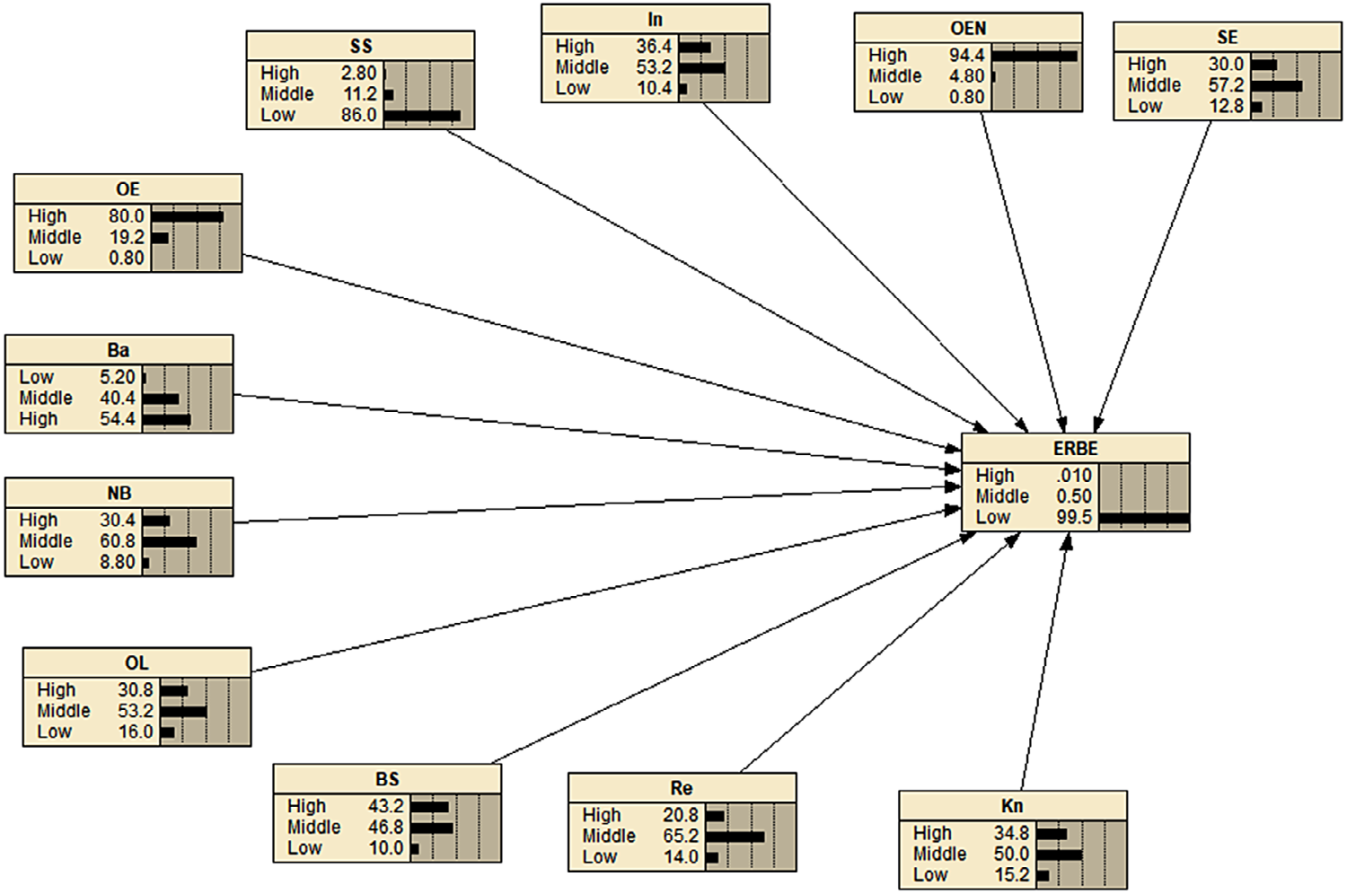
Probability distribution for the Bayesian network association of performance of ERBE. The figure was plotted by Netica. In, Intention; SS, Social Support; OL, Observational learning; Re, Reinforcement; Ba, Barriers; OEN, outcome expectancies; OE, Outcome expectations, SE, Self-efficacy; NB, Normative beliefs; BS, Behavioral skills, Kn, knowledge; ERBE, Ergonomic Behavior

**Fig. 3:**
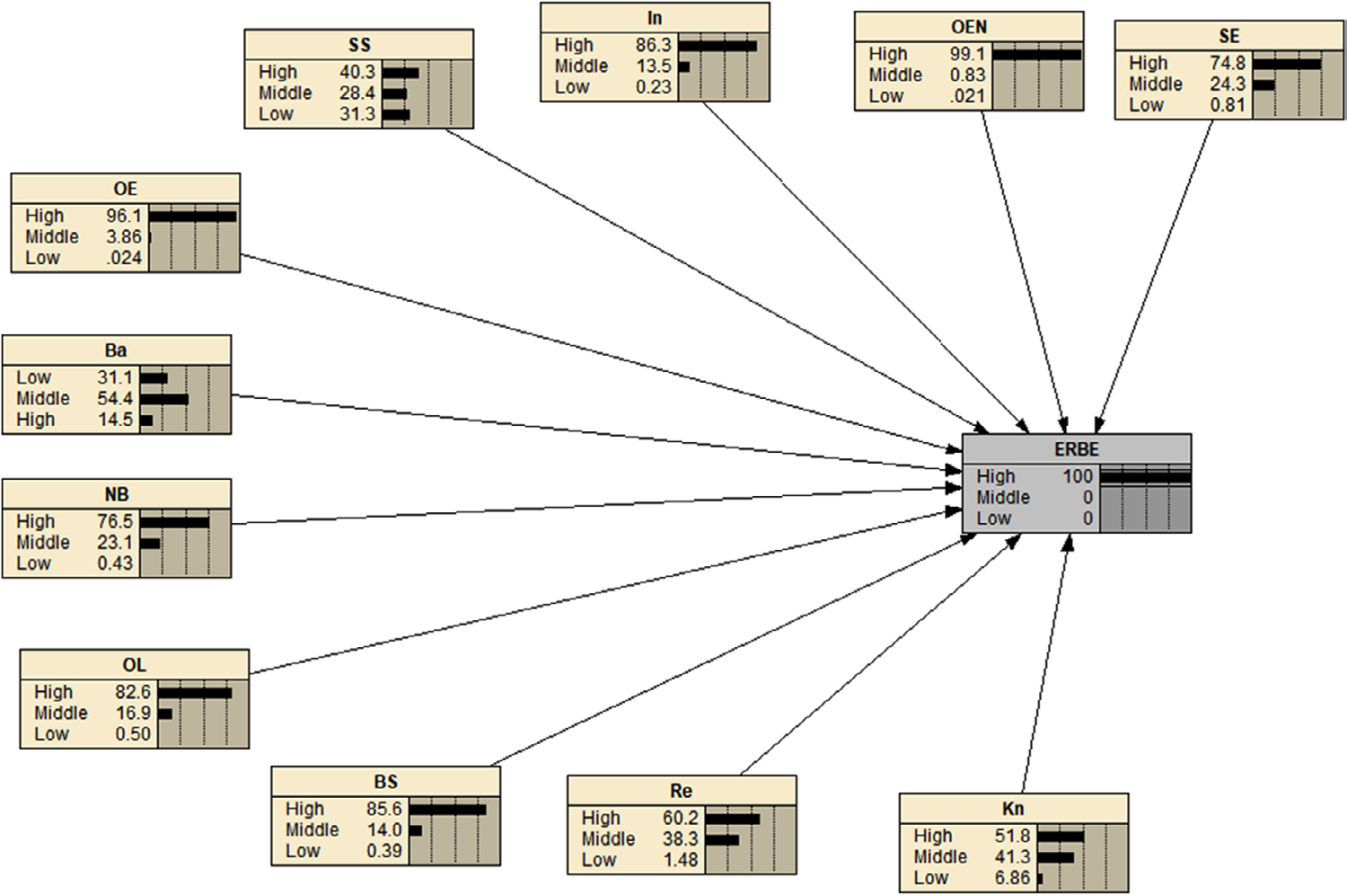
Distributing the conditional probability when ERBE 100 is deemed favorable. In, Intention; SS, Social Support; OL, Observational learning; Re, Reinforcement; Ba, Barriers; OEN, Outcome Expectancies; OE, Outcome Expectations, SE, Self-efficacy; NB, Normative Beliefs; BS, Behavioral skills, Kn, knowledge; ERBE, Ergonomic Behaviors

We constructed the BN model for predicting ERBE based on SCT by identifying the relevant variables and their relationships. The target node was ERBE, and the co-constructs of the theory were the effective factors. We then parameterized the network by assigning weights to each of the parameters and quantified the model. This allowed us to predict the probability of ERBE given the values of the input variables, which can be useful in designing interventions to improve ergonomic behaviors in the workplace. BN serve as visual depictions that illustrate the directional connections between variables, determined by conditional probabilities. The nodes within these networks represent variables and can encompass various potential states, such as high, medium, or low. Associations between variables are depicted through directed edges, with the edges pointing from the independent (parent) variable to the dependent (child) variable [33, 34] (Figs. 2 and 3). One of the key features of BN is their ability to detect even the smallest changes in parent parameters, which distinguishes them from other analytical tools. This sensitivity allows for a detailed analysis of how variations in parent variables affect the child parameter, providing a level of accuracy that is often not achievable through other methods [17, 18].

### Statistical analyses

The questionnaire data were entered into SPSS 21 software, and the information was coded to ensure anonymity and confidentiality. The reliability of the SCT criteria was assessed using Cronbach’s α test. Descriptive statistics methods, such as frequency, percentage, mean, and standard deviation, were employed to characterize the studied population.

To analyze the relationships between the dependent and independent variables, univariate analyses such as independent samples t-tests or ANOVA tests were conducted based on the characteristics of the variables. Additionally, correlation analysis was performed to examine the relationship between the variables of SCT and ERBE.

To examine the factors that influence ERBE, BN analysis was conducted using Netika software. ERBE was considered as the dependent variable (target node), while the constructs of SCT were treated as the independent variables (parent nodes).

## Results

### Participant characteristics

The women who took part in this study can be characterized, on average, as being in the middle-aged category. They tended to have a moderately higher body mass index (BMI) and a moderate level of work experience. A majority of the women in the study were married, with 56.4% reporting no intentional physical activity. Furthermore, only 12% of the participants consumed one or more servings of dairy products per day (Table 1). Table 2 shows different sites of the participants’ bodies with pain experience which they reported.

**Table 1:** Demographic characteristics of the studied women

Variable	Mean ± SD	
Age (year)	35.15 ± 7.99	
BMI (kg/m^2^)	26.60 ± 15.02	
Work experience (year)	8.00 ± 5.91	
	**Number**	**Percent**
Education		
Under diploma	112	44.8
Diploma and upper diploma	138	55.2
Marital status		
Single	57	22.8
Married	145	58.0
Widowed	48	19.2
Finances		
Well	11	4.4
Moderate	125	50.0
Poor	114	45.6
Physical activity		
No purposeful physical activity	141	56.4
Less than 150 min per week	84	33.6
150 min or more	25	10.0
Dairy consumption		
Never	50	20.0
Less than two units	170	68.0
Two or more units	30	12.0

### Common sites affected by chronic musculoskeletal pain

Shoulder, back, and neck regions were identified as the primary sites experiencing persistent pain throughout the last year and week. Furthermore, it was noted that women, in particular, encountered limitations in their daily activities both at home and outside due to pain in these areas. Conversely, ankle pain was reported as less prevalent than in the aforementioned locations, indicating its relatively lower frequency (Table 2).

**Table 2:** Prevalence of musculoskeletal pain during the past year and past week in women

Body part	Past-year musculoskeletal pain N (%)	Past-week musculoskeletal pain N (%)	Prevented you from doing your normal work (at home or away from home) during the past-week N (%)
Neck	169 (67.60)	129 (51.60)	80 (32.00)
Shoulder	185 (74.00)	134 (53.60)	84 (33.06)
Elbow	77 (30.80)	57 (22.08)	41 (16.04)
Wrists/hands	144 (57.60)	121 (48.40)	66 (26.40)
Upper back	150 (60.00)	124 (49.60)	63 (25.20)
Back	170 (68.00)	127 (50.80)	86 (34.40)
Hips/thighs	78 (31.20)	61 (24.04)	42 (16.80)
Knee	133 (53.02)	103 (41.20)	67 (26.70)
Ankles/feet	65 (26.00)	57 (22.80)	38 (15.20)

### Relationship between ERBE with demographic socio-economic factors and SCT constructs

The ANOVA test revealed a significant relationship between ERBE acceptance and both physical activity and dairy consumption. Furthermore, the independent samples t-test indicated a significant relationship between ERBE and education level. Specifically, among women, those who were physically inactive exhibited a lower mean ERBE compared to their physically active counterparts. Additionally, women with a higher level of education and consuming three or more units of dairy products demonstrated a higher mean ERBE in comparison to other groups. However, no statistically significant relationship was found between ERBE and factors such as economic status or marital status in this study (Table 3).

**Table 3:** The Relationship between ERBE and some demographic socio-economic variables

Variable		Ergonomic behavior		F	P value
		Mean	SD		
Physical activity	No purposeful physical activity	3.134	0.987	10.628	< 0.001
	less than 150 min per week	3.678	0.971		
	150 min or more	3.80	0.957		
Dairy consumption	Never	2.960	0.902	9.997	< 0.001
	less than two units	3.400	0.976		
	Two or more units	3.966	1.129		
Level of Education	Under diploma	3.311	0.943	4.472	0.035
	Diploma and Upper diploma	3.464	1.098		
Marital status	Single	3.386	0.977	0.026	0.974
	Married	3.393	0.995		
	Widowed	3.354	1.139		
Finances	Well	3.818	1.328	1.513	0.222
	Moderate	3.424	0.961		
	Poor	3.298	1.038		

The application of Pearson’s correlation coefficient analysis revealed a statistically significant association between ERBE and the constructs of intention, social support, observational learning, and reinforcement. Additionally, Table 4 presents other notable relationships among SCT constructs.

**Table 4:** The Pearson correlation matrix pertaining to the variables of interest

	In	SS	OL	Re	Ba	OE	OEN	SE	NB	BS	kn	Be
In	1											
SS	0.321**	1										
OL	0.518**	0.460**	1									
Re	0.486**	0.498**	0.580**	1								
Ba	-0.211**	-0.276**	-0.096	-0.124	1							
OE	0.012	0.177**	0.008	0.059	0.136*	1						
OEN	0.330**	0.242**	0.237**	0.339**	0.032	0.167**	1					
SE	0.565**	0.300**	0.416**	0.505**	-0.170**	0.023	0.281**	1				
NB	0.267**	0.431**	0.390**	0.439**	-0.110	-0.059	0.207**	0.298**	1			
BS	0.509**	0.367**	0.495**	0.530**	-0.226**	0.059	0.308**	0.612**	0.416**	1		
kn	0.152*	0.024	0.161*	0.005	0.048	0.230**	0.214**	-0.014	0.062	0.041	1	
ERBE	0.431**	0.379**	0.405**	0.270**	-0.289**	-0.061	0.113	0.345**	0.402**	0.345**	0.152*	1

In, Intention; SS, Social Support; OL, Observational learning; Re, Reinforcement; Ba, Barriers; OEN, outcome expectancies; OE, Outcome expectations, SE, Self-efficacy; NB, Normative beliefs; BS, Behavioral skills, Kn, knowledge; ERBE, Ergonomic Behavior

**P < 0.001, *P < 0.05

### Bayesian network analysis of the performance of ERBE

Figure 2 displays the conditional probability table for the structures of SCT in relation to performing ERBE. It is observed that all SCT structures are associated with ERBE. Network analysis reveals that when we anticipate 100% acceptance of the behavior, the barrier structure diminishes, while other SCT constructs demonstrate an increase as depicted in Fig. 3.

Sensitivity analysis aims to explore the connection between a target node’s posterior distribution and its parent nodes. In the context of a BN, this analysis involves modifying the probability distribution of each factor and observing the resulting changes in the target node. The extent of these changes in the target node serves as the sensitivity value for each factor. According to the results of the sensitivity analysis, it was found that OL exhibited the highest sensitivity value (4.08). This finding suggests that if women prioritize and consistently practice ERBE in the workplace, the overall level of ERBE can be significantly improved. Additionally, Table 5 indicates that all constructs of SCT were found to have a notable impact on the level of ERBE.

**Table 5:** Maximum sensitivity values of SCT for the ERBE

Ranking	Node	Sensitivity value
1	Observational learning	4.08
2	Intention	3.82
3	Social Support	3.57
4	Normative Beliefs	2.84
5	Behavioral skills	2.8
6	Self-efficacy	2.78
7	Barriers	2.61
8	Reinforcement	2.22
9	Outcome Expectations	0.856
10	Knowledge	0.4
11	Outcome Expectancies	0.28

## Discussion

Utilizing the BN methodology, this study aimed to uncover the key factors influencing the adoption of ERBE among women employed in assembly line work, leveraging SCT. The findings revealed significant associations between women’s level of education, physical activity, and consumption of dairy products, and their ERBE. Moreover, all variables within the SCT framework demonstrated sensitivity to women’s ERBE, with observational learning, intention, and social support displaying the highest sensitivity compared to other factors.

Our findings indicate a significant prevalence of musculoskeletal pain in the neck, shoulder, and back among WwAL. This is consistent with a recent study conducted by Yang et al., which highlights that electronic assembly line workers face a higher risk of MSDs. This increased risk is attributed to the adoption of poor posture, prolonged periods of sitting or standing, and frequent repetitive actions [29]. Workers who engage in repetitive motions and maintain improper work postures are particularly susceptible to musculoskeletal pain due to the repetitive stress placed on their muscles and joints, leading to overuse injuries [35]. Additionally, the development of muscle fatigue in workers further exacerbates symptoms of musculoskeletal pain [36]. Consequently, it becomes imperative to educate workers on proper postures and provide ergonomic training to mitigate the risk of muscle fatigue and long-term MSDs.

In our study, we observed that individuals who engaged in higher levels of physical activity exhibited a higher average score of ERBE. According to a European study, individuals who engage in low levels of physical activity are more likely to have poor posture. The study found that both a sedentary lifestyle and low physical activity levels have a significant impact on postural parameters [37]. It appears that engaging in physical activity can enhance body condition through the improvement of muscle strength, balance, flexibility, and range of motion [38]. Additionally, a healthier lifestyle in women is associated with increased awareness, motivation and commitment to ERBE. The results of our study indicate that women with a higher level of education exhibit a significantly greater tendency to engage in ERBE. This finding is consistent with the results of a separate Norwegian prospective study, which also identified a statistically significant distinction between groups based on education and physical activity regarding the risk of chronic low back pain [39]. These findings emphasize the importance of education in promoting ergonomic practices among women.

Observational learning displayed the highest degree of sensitivity toward ERBE in this study. Specifically, as observational learning increased, there was a corresponding increase in women’s engagement in ERBE. Observational learning encompasses various impacts, including the acquisition of skills and fostering confidence in one’s capability to execute a particular behavior, known as self-efficacy. Notably, when individuals observe a “coping” model who effectively tackles and overcomes obstacles, their self-efficacy in learning complex new behaviors tends to increase [40]. Intention, as the second component, demonstrated a significant sensitivity to the implementation of ERBE. As intention levels increased, there was a corresponding increase in the frequency of individuals engaging in ergonomic practices. In other comparable instances, intention has proven to be a crucial factor in adopting healthy behaviors [41, 42]. Strong intentions offer more accurate forecasts of behavior and demonstrate greater durability over time, exhibiting increased resilience when subjected to interventions aimed at altering them [43]. Social support emerged as the third construct in our study, demonstrating the highest sensitivity to ERBE. Women who believed they had higher levels of social support were more inclined to engage in ERBE. A systematic review of prospective studies revealed a positive correlation between social support and the adoption of recommended behaviors among adults [44]. Social support has the potential to contribute to the advancement of workers’ healthy behaviors, encompassing exercise, self-care for chronic conditions, and overall health and well-being [45]. Consistent with this, higher levels of social support create an environment that encourages individuals to participate in ERBE by providing emotional support, motivation, practical help, the sharing of knowledge, and fostering self-confidence in their abilities. Additionally, the constructs of normative beliefs, behavioral skills, self-efficacy, perceived barriers, reinforcement, behavioral consequences, knowledge, and outcome expectation value each displayed a degree of sensitivity towards ERBE. Sebastian et al. conducted a study that demonstrated significant associations between all constructs of SCT and behaviors related to a healthy lifestyle [46]. According to this, it appears that when industry authorities endorse and accept ERBE among workers, and when there are minimal barriers to implementing such behaviors in the workplace, coupled with individuals possessing the necessary knowledge and skills, and having confidence in their ability to execute these behaviors, the likelihood of engaging in ergonomic practices increases. The comprehensive examination of SCT constructs in relation to women’s ERBE using BN revealed their collective effectiveness in explaining and predicting such behavior. Additionally, the study found that all constructs displayed varying degrees of sensitivity, indicating their relevance and impact on women’s engagement in ERBE.

### Strengths

The study on WMSDs among WwAL possesses several notable strengths. Firstly, participants from six different industries offer diverse perspectives, enabling a comprehensive examination of the factors influencing WMSDs. Secondly, the study focuses specifically on a vulnerable group, acknowledging and addressing the unique challenges faced by these individuals. Thirdly, the study utilizes a comprehensive tool based on SCT, enabling the identification and assessment of multiple individual, cognitive, and environmental factors that influence behavior. Fourthly, the study utilizes BN Analysis, a powerful statistical technique that enables the identification and modeling of multiple individual, cognitive, and environmental factors. Collectively, these strengths highlight the study’s methodological rigor, its relevance to a vulnerable population, and its comprehensive exploration of influential factors, thereby making a valuable contribution to the field of WMSDs among WwAL.

### Limitations

Given that the data relied on self-reporting, potential biases such as recall and social desirability could have influenced the findings. Additionally, the use of a cross-sectional design limits the ability to establish causal relationships, warranting caution in drawing definitive conclusions. It is recommended to expand the scope of future research to include diverse age and gender groups, as this study exclusively focused on women employed in the assembly line.

## Conclusions

All constructs of SCT prove effective in identifying the factors associated with the involvement of WwAL in ERBE. Given the established correlations between education levels, variables related to a healthy lifestyle, and ERBE, it is clear that these factors should be considered when investigating the adoption of ergonomic practices among this population.

## Data Availability

The datasets generated and/or analyzed during this study are not publicly available due to the presence of sensitive personal information that could compromise confidentiality. Nonetheless, individuals who are interested may inquire about accessing these datasets directly from the corresponding author.

## Abbreviations

ERBE: Ergonomic behaviors
SCT: Social cognitive theory
BN: Bayesian networks
WwAL: women workers on assembly lines
WMSDs: work-related musculoskeletal disorders
MSDs: Musculoskeletal disorders

## References

[1] Sani NT, Widajati N (2021). Factors affecting risk of Musculoskeletal disorders (MSDs) complaints in Spring Production workers. Indian J Forensic Med Toxicol.

[2] Krishnan KS, Raju G, Shawkataly O (2021). Prevalence of work-related musculoskeletal disorders: psychological and physical risk factors. Int J Environ Res Public Health.

[3] Jabbar KM, Gandomi F (2021). The comparison of two corrective exercise approaches for hyperkyphosis and forward head posture: a quasi-experimental study. J Back Musculoskelet Rehabil.

[4] Jia N, Zhang H, Ling R, Liu Y, Li G, Ren Z (2020). Investigation on work-related musculoskeletal disorders—China, 2018 – 2019. China CDC Wkly.

[5] Russo F, Di Tecco C, Fontana L, Adamo G, Papale A, Denaro V (2020). Prevalence of work related musculoskeletal disorders in Italian workers: is there an underestimation of the related occupational risk factors?. BMC Musculoskelet Disord.

[6] Doğrul Z, Mazican N, Turk M (2019). The prevalence of work-related Muskuloskeletal disorders (WRMSDs) and related factors among Occupational Disease Clinic patients. Int Arch Public Heal Community Med.

[7] Bodhare T, Valsangkar S, Bele S (2011). An epidemiological study of work-related musculoskeletal disorders among construction workers in Karimnagar, Andhra Pradesh. Indian J Community Med off Publ Indian Assoc Prev Soc Med.

[8] Etuknwa A, Daniels K, Nayani R, Eib C (2023). Sustainable return to work for workers with mental health and musculoskeletal conditions. Int J Environ Res Public Health.

[9] Price JW (2021). Osteopathic model of the development and prevention of occupational musculoskeletal disorders. J Osteopath Med.

[10] Hoe VCW, Urquhart DM, Kelsall HL, Zamri EN, Sim MR (2018). Ergonomic interventions for preventing work-related musculoskeletal disorders of the upper limb and neck among office workers. Cochrane Database Syst Rev.

[11] Lin S, Tsai CC, Liu X, Wu Z, Zeng X (2022). Effectiveness of participatory ergonomic interventions on musculoskeletal disorders and work ability among young dental professionals: a cluster-randomized controlled trail. J Occup Health.

[12] van de Wijdeven B, Visser B, Daams J, Kuijer PPFM (2023). A first step towards a framework for interventions for individual working practice to prevent work-related musculoskeletal disorders: a scoping review. BMC Musculoskelet Disord.

[13] Okello A, Wafula ST, Sekimpi DK, Mugambe RK (2020). Prevalence and predictors of work-related musculoskeletal disorders among workers of a gold mine in south Kivu, Democratic Republic of Congo. BMC Musculoskelet Disord.

[14] Lee Y-S, Chia M, Komar J (2022). A systematic review of physical activity intervention programs in ASEAN Countries: efficacy and future directions. Int J Environ Res Public Health.

[15] Middleton L, Hall H, Raeside R (2019). Applications and applicability of Social Cognitive Theory in information science research. J Librariansh Inf Sci.

[16] Karen Glanz, Barbara K, Rimer KV. Health Behavior: Theory, Research, and Practice, 5th Edition. 2015. 182–219.

[17] Chen P-C, Chuang C-H, Tu Y-K, Bai C-H, Chen C-F, Liaw M-Y (2015). A Bayesian network meta-analysis: comparing the clinical effectiveness of local corticosteroid injections using different treatment strategies for carpal tunnel syndrome. BMC Musculoskelet Disord.

[18] Rezaianzadeh A, Sepandi M, Rahimikazerooni S (2016). Assessment of Breast cancer risk in an Iranian female population using Bayesian networks with varying node number. Asian Pac J Cancer Prev APJCP.

[19] Niu NN, Davis AM, Bogart LM, Thornhill TS, Abreu LA, Ghazinouri R (2011). Patient Disease perceptions and coping strategies for arthritis in a developing nation: a qualitative study. BMC Musculoskelet Disord.

[20] Antunes MD, Schmitt ACB, Marques AP (2022). Amigos De Fibro (Fibro friends): development of an educational program for the health promotion of fibromyalgia patients. Prim Health Care Res Dev.

[21] Fakhri A, Mohammadi Zeidi I, Morshedi H (2016). Applying the theory of planned behavior to correct posture in operating room staffs. Global J Health Sci.

[22] Bello B, Aminu A, Abdullahi A, Akindele MO, Useh U, Ibrahim AA (2022). Knowledge, attitude, and perception of low back pain and activities that may prevent it among adolescents in Nigeria. Afr Health Sci.

[23] Peterson CL, Evans KD, Axiotis IR (2017). Sonographer scanning practices and musculoskeletal injury: evaluation of an occupational health issue using the health belief model. J Diagn Med Sonography.

[24] Viikari-Juntura E, Martikainen R, Luukkonen R, Mutanen P, Takala E, Riihimäki H (2001). Longitudinal study on work related and individual risk factors affecting radiating neck pain. Occup Environ Med.

[25] Ayu F, Ratriwardhani RA. Relationship of work position with complaints of musculoskeletal disoeders (MSDs) in cracker industrial worker at Kedungdoro village, Sidoarjo. IOP Conference Series: Earth and Environmental Science. 2021;747.

[26] Akbari-Chehrehbargh Z, Tavafian SS, Montazeri A (2022). Influencing factors of the Back Care-related Behavior among Female Schoolchildren: a structural equation modeling. J Educ Community Health.

[27] Hammer C, Degerfeldt L, Denison E (2007). Mechanical diagnosis and therapy in back pain: compliance and social cognitive theory. Adv Physiotherapy.

[28] Chaiklieng S, Suggaravetsiri P (2020). Low back Pain (LBP) incidence, ergonomics risk and workers’ characteristics in relations to lbp in electronics assembly manufacturing. Indian J Occup Environ Med.

[29] Yang F, Di N, Guo WW, Ding WB, Jia N, Zhang H, Li D, Wang D, Wang R, Zhang D, Liu Y, Shen B, Wang ZX, Yin Y (2023). The prevalence and risk factors of work related musculoskeletal disorders among electronics manufacturing workers: a cross-sectional analytical study in China. BMC Public Health.

[30] Hosseini ZS, Tavafian SS, Ahmadi O, Maghbouli R. The ergonomic behaviors evaluation Tool (EBET) based on social cognitive theory for the Assembly Line workers: Development and Psychometric Assessment (under review). BMC Public Health.2023. 10.1186/s12889-024-18738-w PMC11071251 38711084

[31] Moreira S, Criado MB, Santos PC, Ferreira MS, Gonçalves C, Machado J (2022). Occupational health: physical activity, musculoskeletal symptoms and quality of life in computer workers: a narrative review. Healthcare.

[32] Geiker NRW, Mølgaard C, Iuliano S, Rizzoli R, Manios Y, Van Loon LJC (2020). Impact of whole dairy matrix on musculoskeletal health and aging - current knowledge and research gaps. Osteoporos Int.

[33] He Y, Lin Y, He X, Li C, Lu Q, He J (2023). The Conservative management for improving Visual Analog Scale (VAS) pain scoring in greater trochanteric pain syndrome: a Bayesian analysis. BMC Musculoskelet Disord.

[34] Sinclair JE, Mayfield HJ, Short KR, Brown SJ, Puranik R, Mengersen K (2022). A Bayesian network analysis quantifying risks versus benefits of the Pfizer COVID-19 vaccine in Australia. npj Vaccines.

[35] Coenen P, Douwes M, van den Heuvel S, Bosch T (2016). Towards exposure limits for working postures and musculoskeletal symptoms–a prospective cohort study. Ergonomics.

[36] TAIB MFM, Sirat RM (2021). Ergonomics awareness, working posture and muscle fatigue among industry workers and their relationship with musculoskeletal disorders (MSDs) symptoms: a case study. Jurnal Mekanikal.

[37] Sidlauskienė A, Strukcinskiene B, Raistenskis J, Stukas R, Strukčinskaitė V, Buckus R (2019). The association between the level of physical activity with spinal posture and physical fitness parameters in early adolescence. Vojnosanit Pregl.

[38] Zarei H, Norasteh AA, Koohboomi M (2020). The relationship between muscle strength and range of motion in Lower Extremity with Balance and Risk of falling in Elderl. Phys Treatments-Specific Phys Therapy J.

[39] Heuch I, Heuch I, Hagen K, Zwart J-A (2016). Is there a U-shaped relationship between physical activity in leisure time and risk of chronic low back pain? A follow-up in the HUNT study. BMC Public Health.

[40] Van Der Heijden A, Mulder BC, Poortvliet PM, Van Vliet AJH (2017). Social-cognitive determinants of the tick check: a cross-sectional study on self-protective behavior in combatting Lyme Disease. BMC Public Health.

[41] Addis A, Alemnew W, Kassie A, Handebo S (2022). Physical exercise and its associated factors among Ethiopian pregnant women: a cross-sectional study based on the theory of planned behavior. BMC Psychol.

[42] Fishman J, Lushin V, Mandell DS (2020). Predicting implementation: comparing validated measures of intention and assessing the role of motivation when designing behavioral interventions. Implement Sci Commun.

[43] Conner M, Norman P. Understanding the intention-behavior gap: the role of intention strength. Front Psychol. 2022;4249. 10.3389/fpsyg.2022.923464 PMC9386038 35992469

[44] Scarapicchia TMF, Amireault S, Faulkner G, Sabiston CM (2017). Social support and physical activity participation among healthy adults: a systematic review of prospective studies. Int Rev Sport Exerc Psychol.

[45] Hämmig O (2017). Health and well-being at work: the key role of supervisor support. SSM-population Heal.

[46] Sebastian AT, Rajkumar E, Tejaswini P, Lakshmi R, Romate J (2021). Applying social cognitive theory to predict physical activity and dietary behavior among patients with type-2 Diabetes. Heal Psychol Res.

